# Type 2 innate lymphoid cells are not involved in mouse bladder tumor development

**DOI:** 10.3389/fimmu.2023.1335326

**Published:** 2024-01-12

**Authors:** Anna K. Schneider, Sonia Domingos-Pereira, Valérie Cesson, Lenka Polak, Padraic G. Fallon, Jinfang Zhu, Beat Roth, Denise Nardelli-Haefliger, Laurent Derré

**Affiliations:** ^1^ Urology Research Unit and Urology Biobank, Department of Urology, Centre Hospitalier Universitaire Vaudois, Lausanne, Switzerland; ^2^ School of Medicine, Trinity Biomedical Sciences Institute, Trinity College Dublin, Dublin, Ireland; ^3^ Molecular and Cellular Immunoregulation Section, Laboratory of Immune System Biology, National Institute of Allergy and Infectious Diseases, National Institutes of Health, Bethesda, MD, United States

**Keywords:** innate lymphoid cell (ILC), type 2 innate lymphoid cell (ILC2), bladder cancer, tumor microenvironment, MB49 bladder tumor model

## Abstract

Therapies for bladder cancer patients are limited by side effects and failures, highlighting the need for novel targets to improve disease management. Given the emerging evidence highlighting the key role of innate lymphoid cell subsets, especially type 2 innate lymphoid cells (ILC2s), in shaping the tumor microenvironment and immune responses, we investigated the contribution of ILC2s in bladder tumor development. Using the orthotopic murine MB49 bladder tumor model, we found a strong enrichment of ILC2s in the bladder under steady-state conditions, comparable to that in the lung. However, as tumors grew, we observed an increase in ILC1s but no changes in ILC2s. Targeting ILC2s by blocking IL-4/IL-13 signaling pathways, IL-5, or IL-33 receptor, or using IL-33-deficient or ILC2-deficient mice, did not affect mice survival following bladder tumor implantation. Overall, these results suggest that ILC2s do not contribute significantly to bladder tumor development, yet further investigations are required to confirm these results in bladder cancer patients.

## Introduction

Bladder cancer ranks as the 10^th^ most common cancer worldwide (1). The risk of developing a bladder cancer increases with age, smoking, and exposition to certain chemicals (1–3). Urothelial carcinoma represents 90% of bladder cancer cases and can be classified into two main subtypes: non-muscle invasive bladder cancer (NMIBC), confined to the urothelium or lamina propria, and muscle-invasive bladder cancer (MIBC), characterized by invasion into the detrusor muscle or adjacent tissues, with potential metastasis (4, 5). The gold-standard treatments for NMIBC and MIBC are intravesical Bacillus Calmette Guérin (BCG) instillations and cystectomy with or without chemotherapy, respectively. These treatments are limited by significant side effects and failure highlighting the need to investigate novel therapeutic targets to improve the disease management (6).

Identified a decade ago, innate lymphoid cells (ILCs) are viewed as the innate counterpart of CD4 T helper (T_H_) cells (7). They are lymphocytes lacking antigen receptor and classical phenotypic markers for lymphoid and myeloid cells yet exhibit similar functions as T_H_ cells (7, 8). Among the different ILC subsets (9), type 2 innate lymphoid cells (ILC2) are considered as the innate counterpart of T_H_2 cells, and are characterized by their ability to produce type 2 cytokines including IL-4, IL-5 and IL-13 (7, 8). They express the transcription factors GATA-binding protein 3 (Gata3) and retinoic acid receptor-related orphan receptor alpha (RORα), which play crucial roles in their development and function (10–12). ILC2s are generally tissue-resident cells particularly enriched in mucosal and barrier tissues such as the skin, intestine, and lung (8, 13), and rapidly respond to environment changes by sensing factors including neuropeptides, cytokines and alarmins such as IL-25, IL-33 and thymic stromal lymphopoietin released by epithelial and/or myeloid cells under infection or tissue damage (14, 15). While ILC2s are crucial for host defense and tissue homeostasis, their dysregulation has been implicated in inflammatory diseases and allergic responses, including asthma where ILC2s promote airway inflammation leading to airway hyperresponsiveness (16, 17).

Emerging evidence suggests that ILCs, including ILC2s, play significant roles in shaping the tumor microenvironment and anti-tumor immune response (18–21). Tumor-infiltrating ILC2s have been reported to exert anti-tumor activity through direct interaction with T cells (22) or by inducing the recruitment of innate immune cells to the tumor such as eosinophils and dendritic cells, thereby improving T cell-mediated anti-tumor response in melanoma and pancreatic ductal adenocarcinoma (23, 24). However, the presence of ILC2s within the tumor has also been associated with a diminished anti-tumor response (25). Notably, ILC2s have been reported to exert pro-tumoral role by limiting the anti-tumor functions of natural killer cells, promoting tumor burden (26). In addition, ILC2-expressed chemokines and cytokines, particularly CXCL2 and IL-13, have been implicated in the recruitment of immunosuppressive cells such as neutrophils or myeloid-derived suppressor cells (MDSC) into the tumor microenvironment, as observed in hepatocellular carcinoma, breast cancer, and colorectal cancer (27–29). In bladder cancer, our group recently showed that ILC2s detected in the urine of NMIBC patients undergoing BCG treatment, were positively correlated with the frequency of urinary MDSCs and linked to bladder tumor recurrence (30), suggesting a key role of ILC2s in bladder cancer. Overall, the role of ILC2s in cancer is still under debate and seems to depend on the studied model and the type of tumor. Thus, better understanding of their contribution in bladder tumor development is needed before harnessing them for immunotherapy.

In this study, we investigated the involvement of ILC2s in bladder tumor using mouse models. We found the mouse bladder as being particularly enriched in ILC2s at steady state, but their number did not change upon tumor growth. In addition, targeting ILC2s using neutralizing antibodies or different transgenic mouse models did not impact mice survival following bladder tumor challenge. Overall, these data suggest that ILC2s do not contribute significantly to bladder tumor development.

## Results

### High frequency of ILC2s in the murine bladder

**Figure 1:**
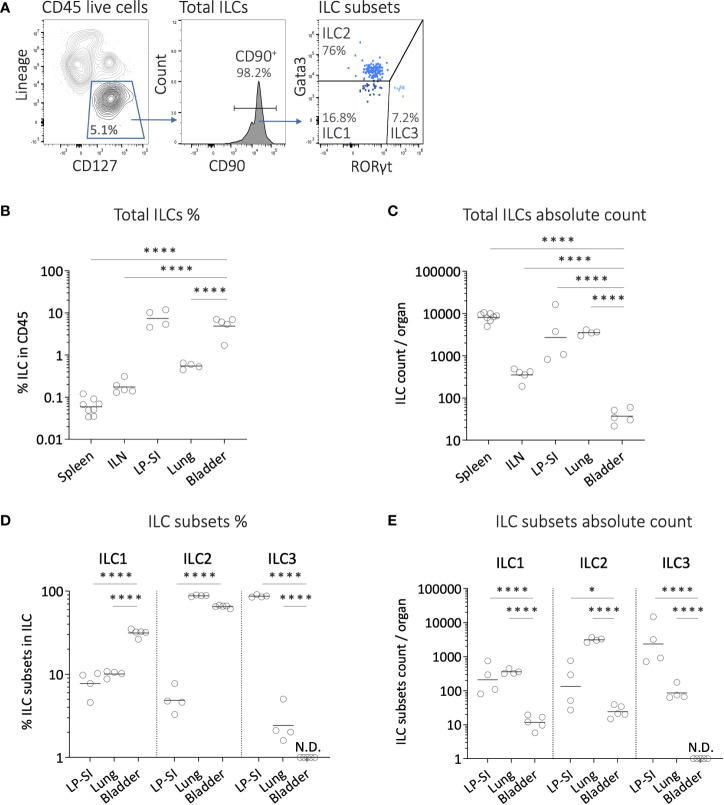
Innate lymphoid cell subsets infiltration in the mouse bladder at steady state. **(A)** Representative example of the gating strategy for analyzing ILC subsets in a bladder from wild-type mouse. After doublets and dead cell exclusion, total ILCs were identified as CD45^+^lineage^neg^CD127^+^CD90^+^ cells (lineage markers including CD3, CD19, FCeRI, CD5, TER119, GR-1, CD11c, F4/80, CD49b, CD11b, TCRγδ, TCRβ, CD8, B220). ILC1s, ILC2s and ILC3s were subsequently identified as Gata3^neg^RORγt^neg^, Gata3^+^RORγt^neg^ and Gata3^neg^RORγt^+^, respectively. **(B)** Frequencies of ILCs in CD45^+^ cells, **(C)** and number of ILCs per organ quantified from spleen, iliac lymph nodes (ILN), lamina propria of the small intestine (LP-SI), lung, and bladder. **(D)** Frequencies of ILC subsets in total ILCs, and **(E)** number of ILC subsets per organ quantified from LP-SI, lung, and bladder. Horizontal lines represent the geometric mean, N.D. not detected. Statistical significances were determined on log-transformed data by parametric one-way ANOVA followed by Bonferroni’s test comparing each condition to bladder. * p ≤ 0.05; **** p ≤ 0.0001.

To assess the presence of ILCs in the bladder, we conducted a comparative analysis of ILC infiltration in different organs at steady state (i.e., without tumor). Flow cytometry analysis was performed to evaluate the infiltration of total ILCs, ILC1s, ILC2s and ILC3s (**Figure 1A**, **Supplementary Figure 1A**) in the bladder, the spleen and iliac lymph nodes (bladder draining lymph nodes; ILN). As control, we also included the lung, and lamina propria of the small intestine (LP-SI), two well-known homing sites for ILC2s and ILC3s, respectively (**Supplementary Figure 1A**) (13, 16). Interestingly, the frequency of ILCs among CD45^+^ cells in the bladder was comparable to that observed in the LP-SI, and significantly higher than in the lung, spleen, and ILN (**Figure 1B**). However, due to a limited infiltration of total immune cells into the bladder at steady state (**Supplementary Figure 1B**), the absolute number of total ILCs infiltrating the bladder was significantly lower compared to the other organs (**Figure 1C**). Among bladder ILC subsets, we surprisingly found a very high frequency of ILC2s (ca. 70% of total ILCs), comparable to that in the lung (**Figure 1D**). In addition, while the frequency of ILC3s was barely detectable, the proportion of ILC1s was higher in the bladder compared to both the lung and lamina propria. Overall, ILC2s represented the predominant ILC subset in the bladder (**Supplementary Figure 1C**). However, the absolute number of ILC subsets infiltrating the bladder was significantly lower compared to the lung and LP-SI (**Figure 1E**).

### Bladder-infiltrating ILC2s remained stable upon tumor growth, while ILC1 absolute number increased

**Figure 2:**
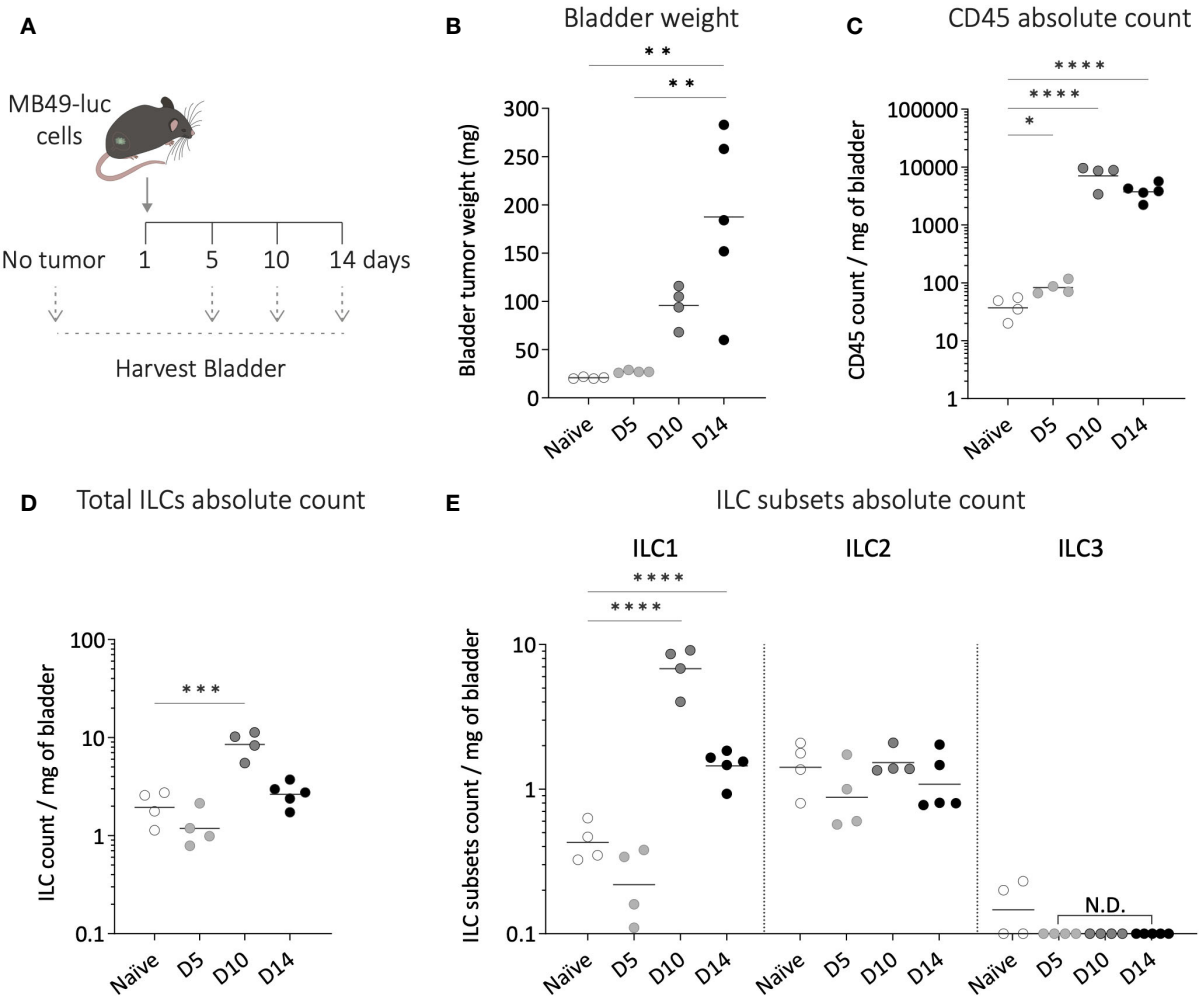
Dynamics of bladder infiltrating ILC subsets during tumor growth. **(A)** Luciferase-expressing MB49 tumor cells (MB49-luc) were instilled in wild-type mice on day 1 and bladders were harvested at different time points of the tumor growth (day 5-10-14) for ILC subsets flow cytometry analysis. **(B)** Bladder weight at each time point of the tumor growth. Number per mg of bladder of **(C)** total immune cells, **(D)** ILCs, **(E)** and ILC subsets along tumor growth. Horizontal lines represent the mean **(B)** or geometric mean **(C–E)**, N.D. not detected. Statistical significances were determined by parametric one-way ANOVA followed by Bonferroni’s test. Comparisons are shown between each time point **(B)**, or on log-transformed data for each time point versus naïve **(C–E)**. * p ≤ 0.05; ** p ≤ 0.01; *** p ≤ 0.001; **** p ≤ 0.0001.

To investigate the potential role of ILC2s in bladder tumor development, we next characterized the changes in bladder ILC infiltration throughout tumor growth, using the orthotopic murine MB49 bladder tumor model. Luciferase-expressing MB49 tumor cells were used to monitor tumor establishment and growth via *in vivo* bioluminescent imaging. Bladder cells obtained from mice at different time points after bladder tumor implantation (day 5, 10 and 14) were analyzed for ILC subsets and compared to bladder cells recovered from mice without tumor (**Figure 2A**). Of note, due to a high variability in bladder weight during tumor growth (**Figure 2B**), immune cell absolute numbers were normalized to the bladder weight at each time point. We observed a significant increase in the absolute number of total immune cells infiltrating the bladder during tumor growth (**Figure 2C**). ILC infiltration displayed a significant increase only on day 10 of tumor development (**Figure 2D**). Compared to the fold-increase in the total population of immune cells, ILCs did not represent the main subset expanding significantly with tumor growth.

Among the different ILC subsets, the bladder exhibited comparable infiltration levels during steady state and early tumor growth (D5), with ILC2s remaining the most abundant subset (**Figure 2E**). However, as the tumor grew, the number of ILC1s per milligram of bladder significantly increased, reaching a ca. 10-fold increase on day 10, this subpopulation becoming the predominant ILC subset in the bladder. In contrast, the level of ILC2s remained stable throughout tumor growth, while ILC3s remained almost undetectable (**Figure 2E**).

We also evaluated the functional status of ILC2s from the bladder by assessing their capacity to secrete IL-13 (20). Upon *in vitro* stimulation, ILC2s from both bladder tumor-free and tumor-bearing mice secreted IL-13, with a significant increase in secretion levels observed in the presence of the tumor (**Supplementary Figure 2**). These results suggest that bladder-infiltrating ILC2s are functional, even in the presence of bladder tumor.

### Inhibiting ILC2 activation and function did not alter mice survival after bladder-tumor challenge

**Figure 3:**
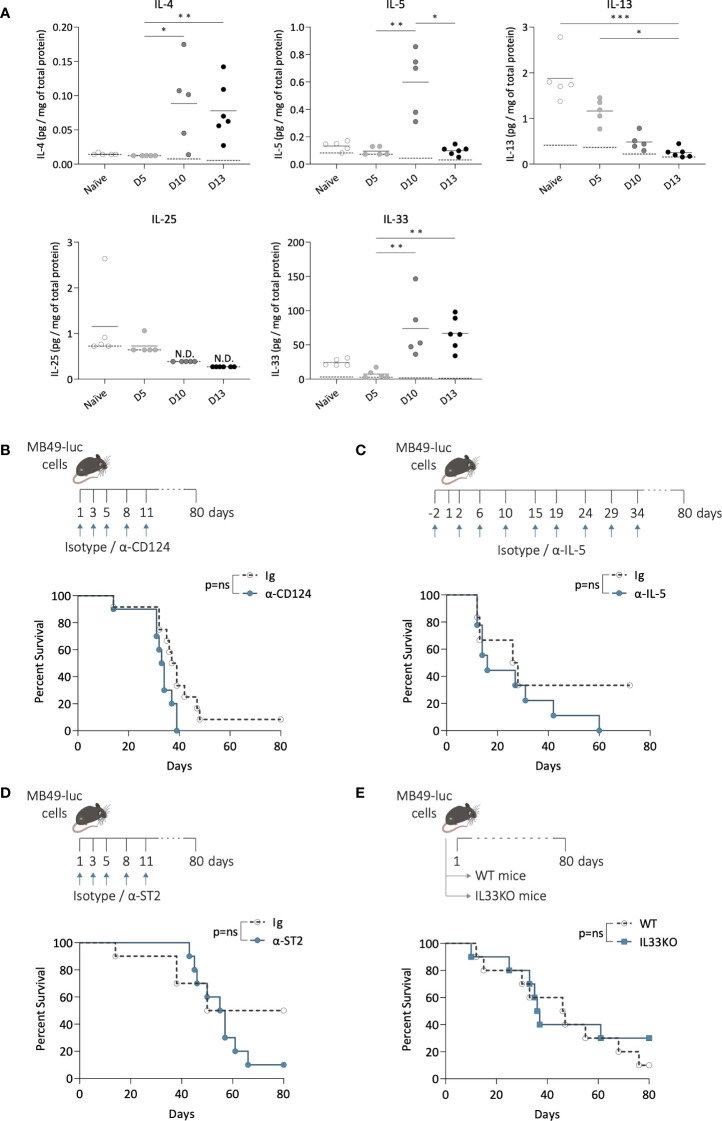
Inhibition of ILC2 activation and function during bladder tumor challenge. **(A)** Level of IL-4, IL-5, IL-13, IL-25 and IL-33 per mg of total protein in bladders of mice without tumors (naïve) and at indicated time point after tumor instillation (day 5, 10, and 13). Horizontal solid lines represent the mean, and the dotted lines represent the limit of detection, N.D. not detected. Statistical significances were determined by one-way ANOVA followed by Dunn’s test comparing the different time points for each cytokine. **(B–D)** Mice were treated i.p. with blocking antibodies or isotypes control at indicated days (arrows) and instilled with MB49 tumor cells in the bladder at day 1. **(B)** Survival curves of mice treated with α-CD124 (n=10) or isotype control (Ig; n=12), or **(C)** with α-IL-5 (n=9) or isotype control (Ig; n=6), or **(D)** with α-ST2 (n=10) or isotype control (Ig; n=10). **(E)** Survival curves of wild-type (WT; n=10) and IL-33-deficient (IL33KO; n=10) mice after bladder tumor implantation. Log-rank test was performed as statistical analysis for survival curves. * p ≤ 0.05; ** p ≤ 0.01; *** p ≤ 0.001; ns: not significant.

To investigate whether ILC2s may alter the bladder tumor development, we studied the effects of targeting ILC2 pathways on tumor growth and mice survival. We first quantified the concentrations of cytokines known to be involved in ILC2s activation and function (20) by luminex assay in bladder samples from tumor-free mice (naïve) and during tumor growth (day 5, 10, and 13) (**Figure 3A**). At steady state, IL-13 and IL-33 were present in the bladder, while IL-4 was undetectable, and IL-5 and IL-25 levels were close to the detection limit. During tumor growth, although IL-13 levels substantially dropped to a very low level, and IL-25 remained very low, IL-4, IL-5, and IL-33 levels significantly increased, with a peak observed only on day 10 for IL-5, suggesting that IL-4, IL-5, and IL-33 related pathways may be activated along bladder tumor growth. Thus, to inhibit ILC2 functions, mice were treated with a blocking antibody targeting IL-4Rα (CD124) (31–33). No differences compared to the control mice were observed in tumor growth (**Supplementary Figure 3A**), or in mouse survival (**Figure 3B**). Then we inhibited ILC2 functions by targeting IL-5 using a blocking antibody (34). Similar to the blocking of IL-4Rα, no differences in tumor growth (**Supplementary Figure 3B**) or mouse survival (**Figure 3C**) were observed when compared to the control mice.

We next inhibited ILC2 activation by targeting IL-33Rα (ST2). IL-33 signaling through its receptor ST2 has been reported as a key pathway for inducing the secretion of type 2 cytokines by ILC2s, and neutralizing ST2 *in vivo* significantly limited ILC2 activation (35–37). We first confirmed the high expression of ST2 by bladder ILC2s compared to ILC1s and 3, which remained stable along the bladder tumor growth (**Supplementary Figure 3C**), consistent with previous observations (38) and suggesting that bladder ILC2s may be responsive to IL-33. Mice were then treated with an anti-ST2 blocking antibody (**Figure 3D**). No significant differences in tumor growth were observed compared to the control mice (**Supplementary Figure 3D**), and no changes in survival were detected (**Figure 3D**). Similar results were obtained when using IL-33-deficient mice (**Supplementary Figure 3E**, **Figure 3E**), which are known to exhibit reduced ILC2 infiltration in tissues and/or less functionally competent ILC2s (39–41). Overall, these data suggest that ILC2 may not play a critical role in bladder tumor development.

### Depletion of ILC2s did not impact mice survival after bladder-tumor challenge

**Figure 4:**
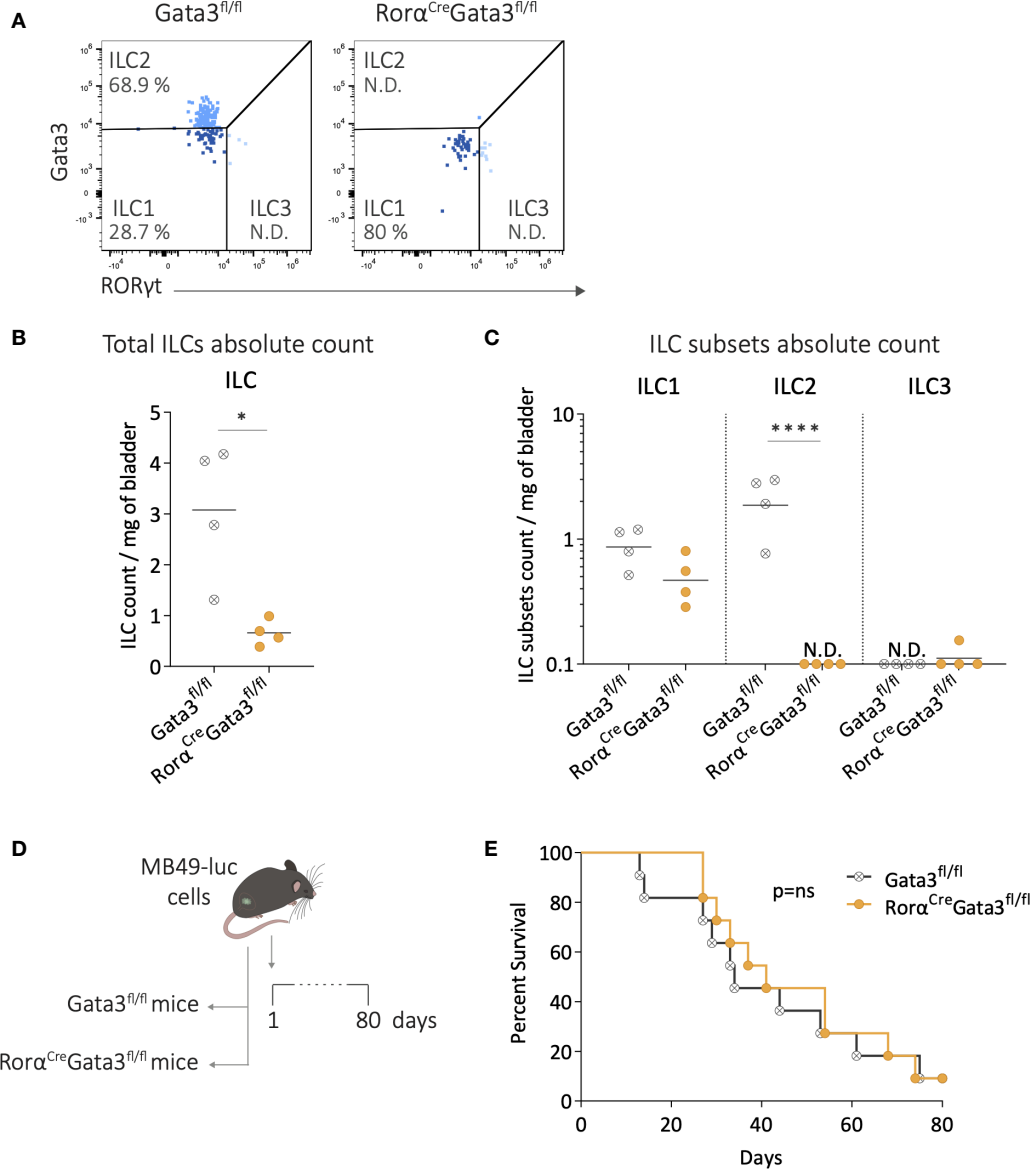
Depletion of ILC2s during bladder-tumor challenge. **(A)** Representative flow cytometry plots of ILC subsets from bladders of Gata3^fl/fl^ and Rorα^Cre^Gata3^fl/fl^ mice at steady state. Number per mg of bladder of **(B)** total ILCs, and **(C)** ILC subsets in Gata3^fl/fl^ and Rorα^Cre^Gata3^fl/fl^ mice at steady state. Horizontal lines represent the mean **(B)** or geometric mean **(C)**, N.D. not detected. **(D)** Luciferase-expressing MB49 tumor cells (MB49-luc) were instilled in Gata3^fl/fl^, and Rorα^Cre^Gata3^fl/fl^ mice on day 1 followed by survival monitoring. **(E)** Survival curves of Gata3^fl/fl^ (n=11) and Rorα^Cre^Gata3^fl/fl^ (n=11) mice after MB49 bladder tumor instillation. Statistical significances were determined by parametric **(B)** t test or **(C)** one-way ANOVA followed by Bonferroni’s test comparing the infiltration between Gata3^fl/fl^ and Rorα^Cre^Gata3^fl/fl^ mice. Log-rank test was performed as statistical analysis for survival curves. * p ≤ 0.05; **** p ≤ 0.0001.; ns: not significant.

We finally studied bladder tumor development in mice lacking ILC2s, using Rorα^Cre^Gata3^fl/fl^ ILC2-deficient mice (42). We first evaluated the extent of ILC2 deficiency in these mice compared to their littermate controls (Gata3^fl/fl^) under steady-state conditions (**Figure 4A**). Flow cytometry analysis revealed a significant reduction in the number of ILCs in the bladders of Rorα^Cre^Gata3^fl/fl^ mice compared to the littermate controls (**Figure 4B**). Among the different ILC subsets, ILC2s were nearly undetectable in the bladders of ILC2-deficient mice, while the infiltration of ILC1s and ILC3s remained unchanged compared to the littermate controls (**Figure 4C**). In addition, there were no differences in the T-cell compartment, CD11b, and NK cells between ILC2-deficient mice and littermate controls (**Supplementary Figure 4A**). These data confirmed the specific depletion of ILC2s in Rorα^Cre^Gata3^fl/fl^ mice. Then tumor was implanted in the bladder of ILC2-deficient mice and littermates (**Figure 4D**). The absence of ILC2s did not affect bladder tumor development (**Supplementary Figure 4B**) or the mice survival compared to littermate Gata3^fl/fl^ (**Figure 4E**). Altogether, these results suggest that bladder ILC2s do not play a central role in bladder tumor development.

## Discussion

In this study, we investigated the involvement of ILC2s in the bladder during tumor growth using mouse models. We found the mouse bladder as being particularly enriched in ILC2s at steady state, similar to the enrichment described in barrier tissues such as the lung. ILC subsets in the bladder are poorly described in the literature. Scharff et al. (43) are among the first to report bladder-resident ILC populations. Together with more recent studies (38, 44), they reported an increase of ILC3s in mouse bladders following uropathogenic Escherichia coli infection and highlighted their importance against the infection. Under steady-state conditions, they also suggested that ILC2s were the main ILC subset in mouse bladders, consistent with our own results (38, 44). Nevertheless, while the levels of ILC subsets differed between these studies, they consistently reported higher levels of bladder-infiltrating ILC3s at steady state compared to our results (38, 44). These differences could be potentially explained by differences in the lineage and/or gating strategies. The lack of specific markers for ILC subsets poses a challenge, as an overly permissive lineage would overestimate ILC subsets, whereas an overly restrictive lineage would underestimate them. Based on the differences observed in the literature regarding ILC3 subset, our lineage approach may be considered relatively restrictive. However, we were able to properly detect ILC3s in the lamina propria of the small intestines, which is a well-known homing sites for ILC3s, arguing that our lineage seems accurate and comprehensive. Further investigations are warranted to clarify this point.

In our study, we investigated the potential involvement of ILC2s in bladder tumor development using various approaches. Neutralizing ILC2 activation and function in mice through antibody blockade during bladder tumor growth suggested that ILC2 may not play a critical role in bladder tumor development. Nevertheless, it should be considered that all the factors involved in ILC2 pathways were not specifically targeted by the blocking antibodies used in our study, potentially leading to a partial neutralization of ILC2 activation and function. In addition to cytokine secretion and soluble factors, ILC2s can exert their functions through cell-to-cell contact (21), suggesting that direct interactions with other immune cells may contribute to the overall effects of ILC2s in the tumor microenvironment. To limit this bias in our investigations, we completed our study on ILC2s in bladder tumor development using transgenic ILC2-deficient mice. However, the absence of ILC2s in these mice did not affect the mice survival following bladder tumor challenge, in line with the results obtained when blocking IL-4/IL-13 or IL-5 signaling pathways, or IL-33/ST2 axis. Overall, these results suggest that ILC2 may not contribute significantly to bladder tumor development.

Although the involvement of ILC2s in mouse bladder tumor growth appeared marginal, our results have to be confirmed in bladder cancer patients. A recent study (45) reported ILC1s and ILC2s as the most frequent subsets infiltrating muscle invasive bladder tumors in patients, and low levels of ILC3s. Similarly to what we observed during mouse bladder tumor growth, higher levels of ILC1s were associated with more advanced tumor stages suggesting a potential role in disease progression. Notably, tumor-infiltrating ILC1s displayed an exhausted T_H_17-like phenotype suggesting potential functional impairment of ILC1s within the bladder. Additional studies are therefore required to elucidate the role of ILC1s in bladder tumor. Moreover, we previously reported higher levels of circulating ILC2s in MIBC patients at the time of the cystectomy compared to NMIBC patients or healthy donors (30). In the context of BCG therapy, our previous investigations in patients also suggested a potential role for ILC2s in bladder cancer recurrence, possibly through IL-13 secretion and MDSC recruitment (30). BCG therapy elicits a robust release of cytokines and chemokines within the bladder, which may potentially impact the role of ILC2s in the bladder tumor microenvironment as ILC2 plasticity and sensitivity to microenvironmental cues render their responses highly context-specific (46). In addition, ILC2s are known for their production of type 2 cytokines and their role in promoting T_H_2 immune responses (47), thus they might be of important in the T_H_1/T_H_2 balance and in influencing treatment response during BCG therapy (48–51).

In summary, while ILC2s have been implicated in promoting tumor growth and immunosuppression in other cancer models, our study in bladder cancer suggests that ILC2-related pathways may not be the principal drivers of tumor development. However, further investigations of ILC infiltration in bladder tissues from spontaneous bladder tumor model and bladder cancer patients, with the assessment of effector cytokine secretion and activation or exhaustion markers, are warranted to clarify the involvement of ILC subsets in bladder cancer, including over the BCG treatment course.

## Materials and methods

### Tumor cell line

MB49 cell line is derived from a carcinogen induced urothelial carcinoma in male C57Bl/6 mice (52). The cell line was provided by Prof. A. Loskog (Uppsala University, Sweden) and transfected with lentiviral vectors encoding for firefly luciferase (provided by Prof. D. Trono, EPFL, Switzerland) to generate luciferase (luc)-expressing MB49 cells (53). MB49-luc cells were cultured at 37°C in a humidified incubator with 5% CO2, in DMEM containing glutamax-1 and sodium pyruvate complemented with 10 mM HEPES, 100U/mL of penicillin-streptomycin, and 10% FCS.

### Mouse models

7 to 10-week-old female C57BL/6JOlaHsd mice were purchased from Envigo. Roratm1(cre)Ddmo mice (54) provided by Prof. P.G. Fallon (Trinity college, Ireland) were crossed with Gata3fl/fl mice (Taconic line 355) (55) ordered from the NIAID-Taconic repository, to generate Rorα^Cre^Gata3^fl/fl^ mice (42). Il33^gfp/gfp^ mice obtained through the RIKEN Center for Developmental Biology (Acc. No. CDB0631K) (56) were provided by Prof. S. Luther (University of Lausanne (UNIL), Switzerland). All mice were bred and housed at the animal facility of the University of Lausanne. All *in vivo* experiments were conducted in accordance with the Swiss law and approval of the Cantonal Veterinary Office of Canton de Vaud, Switzerland.

### MB49 orthotopic bladder tumor model

As previously described (57, 58), bladders of deeply anesthetized female mice were treated with 100 μL of ethanol 22% for 15 min, followed by the intravesical instillation of 500 000 MB49-luc cells resuspended in 50 μL of Hanks’ Balanced Salt Solution (HBSS) using catheters. To monitor tumor growth, mice were administered an intraperitoneal injection of D-luciferin (150 μg/g of body weight; Biosynth) 15 min before measuring bioluminescence of MB49-luc tumor cells with a Xenogen imaging system (Xenogen/IVIS lumina III). In order to avoid bias in the comparison of tumor growth data, bioluminescence imaging was not performed as soon as one mouse died in either of the tested groups. In addition, after the first 3 weeks of tumor growth, bioluminescence is often loss, due to presence of necrotic tumors and/or low tumor-perfusion, preventing tumor-penetration of the injected luciferin (53, 59), thereby tumor growth was monitored by palpation, hematuria, and overall health status of the mice.

### Preparation of mouse organ cell suspensions

Mice were sacrificed by CO2 inhalation and organs were harvested. Single-cell suspensions from spleen were obtained through mechanical dissociation by scratching the spleen onto a 70-μm filter, followed by a red blood cell lysis buffer treatment, and subsequently filtered through a 40-μm filter. Single-cell suspensions from lymph nodes were obtained through mechanical dissociation by scratching the lymph nodes onto a 40-μm filter. Bladder and lungs were minced and digested in 2 mL/organ of digestion buffer containing 1 mg/mL collagenase/dispase or collagenase D, 0.1 mg/mL DNase I, and FCS 5% in Iscove’s modified Dulbecco’s medium with glutamax-1, for 45 min at 37°C in a rotating shaker. After stopping the digestion with FACS buffer (PBS, 2 mM EDTA, and 0.2% BSA), bladder cell suspensions were scratched onto a 40-μm filter, while lung cell suspensions were scratched onto a 70-μm filter and subsequently filtered through a 40-μm filter. Cell suspensions were centrifuged for 10 min at 1000 rpm at room temperature and lung pelleted cells were then treated with red blood cells lysis buffer. All cell suspensions from spleen, lymph nodes, bladder and lung were resuspended in DMEM containing glutamax-1 and sodium pyruvate complemented with 10 mM HEPES, 100 U/mL of penicillin-streptomycin, and 10% FCS. Mice without tumors and tumor-bearing mice at day 5 were pooled by 2 to 3 before processing the organs to increase the number of cells recovered from bladder and ILN.

For the small intestines, fat and Peyer’s patches were removed from the intestine walls, and intestinal contents were removed by flushing with cold PBS. Small intestines were opened longitudinally to remove the mucus then cut in 1-cm pieces. The pieces were shaken several times in PBS to remove any residual intestine contents. Subsequently, intestinal pieces were incubated in 20 mL of intestine epithelial fraction buffer containing HBSS, EDTA 5mM, dithiothreitol 1 mM, and FCS 5% for 20 min at 37°C in a shaker at 250 rpm. After 20 min the supernatant with the epithelial fraction was discarded and the fragments rinsed with PBS. This step was repeated twice. The intestinal fragments were then collected and digested a first time in 10 mL of digestion buffer with RPMI medium, Collagenase/dispase 1 mg/mL, DNase 20 μg/mL, and FCS 10% for 45 min at 37°C in a shaker at 250 rpm. After 45 min, the supernatant was collected through a 70-μm filter and resuspended with PBS-FCS 2%, and the intestinal fragments were digested a second time for 30 min at 37°C in a shaker at 250 rpm. After 30 min, the supernatants were collected through a 70-μm filter, centrifuged for 15 min at 400 x g and resuspended with PBS-FCS 2%.

### Flow cytometry analysis

Recovered cells from mouse samples were stained and analyzed by flow cytometry. The following antibodies were used for staining in mouse samples: anti-GATA3 (BD Biosciences, Cat# 563349, BV421, clone L50-823), anti-RORγt (Thermo Fisher Scientific, Cat# 61-6981-80, PE/eF610, clone B2D), anti-CD90.2 (Thermo Fisher Scientific, Cat# 47-0902-82, APCeF780, clone 53-2.1), anti-CD127 (Biolegend, Cat# 135012, APC, clone A7R34), anti-CD45 (Biolegend, Cat# 103128, AF700, and BD Biosciences, Cat# 564279, BUV395, clone 30-F11), anti-CD3 (Thermo Fisher Scientific, Cat# 11-0031-85, FITC, and BD Biosciences, Cat# 745836, BB700, clone 145-2C11), anti-CD19 (Thermo Fisher Scientific, Cat# 11-0193-82, FITC, clone 1D3), anti-FCeRI (Biolegend, Cat# 134306, FITC, clone MAR-1), anti-CD5 (Biolegend, Cat# 100605, FITC, clone 53-7.3), anti-TER119 (Biolegend, Cat# 116206, FITC, clone TER-119), anti-GR-1 (Biolegend, Cat# 108405, FITC, clone RB6-8C5), anti-CD11c (Biolegend, Cat# 117306, FITC, clone N418), anti-F4/80 (Biolegend, Cat# 123108, FITC, clone BM8), anti-CD49b (Thermo Fisher Scientific, Cat# 11-5971-85, FITC, and BD Biosciences, Cat# 562453, PE-CF594, clone DX5), anti-CD11b (Biolegend, Cat# 101206, FITC, and BD Biosciences, Cat# 612977, BUV661, clone M1/70), anti-TCRγδ (Thermo Fisher Scientific, Cat# 11-5711-85, FITC, clone GL-3), anti-TCRβ (Biolegend, Cat# 109206, FITC, clone H57-597), anti-CD8 (Biolegend, Cat# 100706, FITC, and BD Biosciences, Cat# 563152, BV605, clone 53-6.7), anti-B220 (Thermo Fisher Scientific, Cat# 11-0452-85, FITC, clone RA3-6B2), anti-CD4 (BD Biosciences, Cat# 612761, BUV737, clone GK1.5), anti-ST2 (Thermo Fisher Scientific, Cat# 25-9335-82, PE/Cy7, clone RMST2-2). For cell surface antigen staining, cells were incubated for 30 minutes at 4°C using antibodies described above, and an amine reactive dye (LIVE/DEAD™ Fixable Aqua Dead Cell Stain Kit, Thermo Fisher Scientific) was used for dead cell exclusion according to the manufacturer’s instructions. To block unspecific binding of antibodies, mouse serum was used. After extracellular staining, cells were fixed and permeabilized, and intracellular staining was performed according to manufacturer instruction, using the Foxp3/Transcription Factor Staining Buffer Set (Thermo Fisher Scientific). Sample acquisition was performed on Cytoflex LX1 (Beckman Coulter) or LSRFortessa (Becton Dickinson), and data were analyzed using FlowJo software.

### *In vitro* stimulation for IL-13 secretion

Recovered cell suspension from bladder of naïve or bladder tumor-bearing mice were seeded in 24-well plates (between 10^6^ and 2.10^6^ cells per well) and stimulated for 4 h at 37°C with phorbol 12-myristate 13-acetate (PMA) and ionomycin (Cell stimulation cocktail 500X, Thermo Fisher Scientific, Cat# 00-4970-03) or left unstimulated (culture medium only). After 2h of stimulation, brefeldin A and monensin were added in all the wells (Protein Transport Inhibitor Cocktail 500X, Thermo Fisher Scientific, Cat# 00-4980-93) for intracellular retention of the induced cytokine expression. Following stimulation, cells were collected for intracellular staining of ILC panel and IL-13 (Thermo Fisher Scientific, Cat# 46-7133-82, PerCP-eF710, clone eBio13A).

### Cytokines array in mouse bladder during tumor growth

Bladders from tumor-free mice (naïve) and during tumor growth (day 5, 10, and 13) were homogenized in PBS with a cocktail of protease inhibitors containing aprotinin (10 μg/mL, Sigma-Aldrich), leupeptin (10 μg/mL, Tocris), and pepstatin (10 μg/mL, Tocris) using a tissue grinder. Bladders without tumors and 5 days after tumor implantation were pooled by 2 before processing. After homogenization, Triton™ X-100 (Sigma-Aldrich) was added to a final concentration of 1%. Samples were frozen on dry ice, thawed and centrifuge at 10,000 x g for 5 minutes at 4°C to remove cellular debris. Samples were stored at -80°C until further analysis. Sample total protein concentrations were quantified using a Pierce BCA Protein Assay Kit (Thermo Fisher Scientific) following the manufacturer protocol for 96-well plate procedure. Finally, a Luminex assay was done according to the manufacturer instructions (Bio-Plex Pro Mouse Cytokine assays from Bio-Rad) for quantification of the following cytokines: IL-4, IL-5, IL-13, IL-25, IL-33. Samples were analyzed with a Bio-Plex 200 system.

### Blocking antibody treatments in mice

To neutralize IL-4Rα, mice were injected intraperitoneally with blocking antibodies α-CD124 (100 μg/injection/mouse; BD Biosciences, Cat# 552288, clone mIL4R-M1) or rat IgG2a k isotype control (BioXcell, Cat# BE0089, clone 2A3) diluted in PBS at day 1-3-5-8-11 since bladder-tumor implantation. To neutralize IL-5, mice were injected intraperitoneally with blocking antibodies α-IL-5 (300 μg/injection/mouse; BioXcell, Cat# BE0198, clone TRFK5) or rat IgG1a k isotype control (BioXcell, Cat# BE0088, clone HRPN) diluted in PBS at day -2-2-6-10-15-19-24-29-34 and bladder-tumor implantation at day 1. To neutralize IL-33Rα, mice were injected intraperitoneally with blocking antibodies α-ST2 (100 μg/injection/mouse; Biolegend, Cat# 146604, clone DIH4), or rat IgG1 k isotype control (Biolegend, Cat# 400457, clone RTK2071) diluted in PBS at day 1-3-5-8-11 since bladder-tumor implantation.

### Quantification and statistical analysis

Statistical analyses were performed using the GraphPad Prism 9 software as indicated in the figure legends. Recurrence-free and progression-free survival rates were evaluated using Kaplan-Meier estimator.

## Data availability statement

The original contributions presented in the study are included in the article/**Supplementary Material**. Further inquiries can be directed to the corresponding author.

## Ethics statement

The animal study was approved by Swiss Federal Veterinary Office (SFVO). The study was conducted in accordance with the local legislation and institutional requirements.

## Author contributions

AS: Data curation, Formal Analysis, Investigation, Methodology, Writing – original draft. SD-P: Writing – review & editing, Investigation, Methodology. VC: Writing – review & editing, Investigation, Methodology. LP: Investigation, Writing – review & editing. PF: Writing – review & editing, Resources. JZ: Resources, Writing – review & editing. BR: Resources, Validation, Writing – review & editing, Methodology. DN-H: Funding acquisition, Resources, Writing – review & editing, Methodology. LD: Conceptualization, Funding acquisition, Project administration, Resources, Supervision, Validation, Writing – original draft, Writing – review & editing.
