# Supplementary figures and images for: Type 2 innate lymphoid cells are not involved in mouse bladder tumor development
Source: Front Immunol. 2024 Jan 12;14:1335326. doi: 10.3389/fimmu.2023.1335326 (PMC10820705; doi:10.3389/fimmu.2023.1335326)

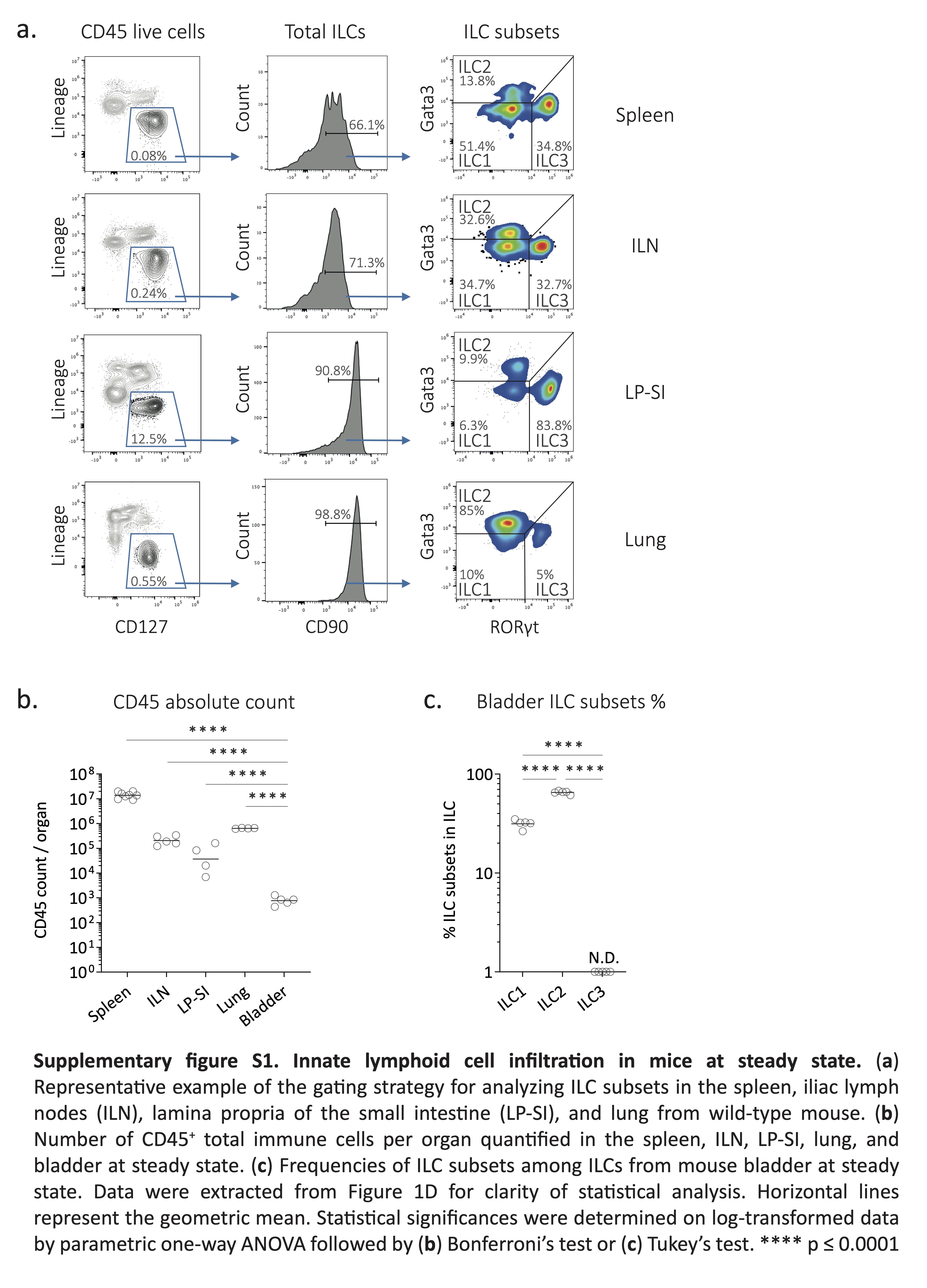

Supplement: Supplementary file 1 [file Image_1.jpg]

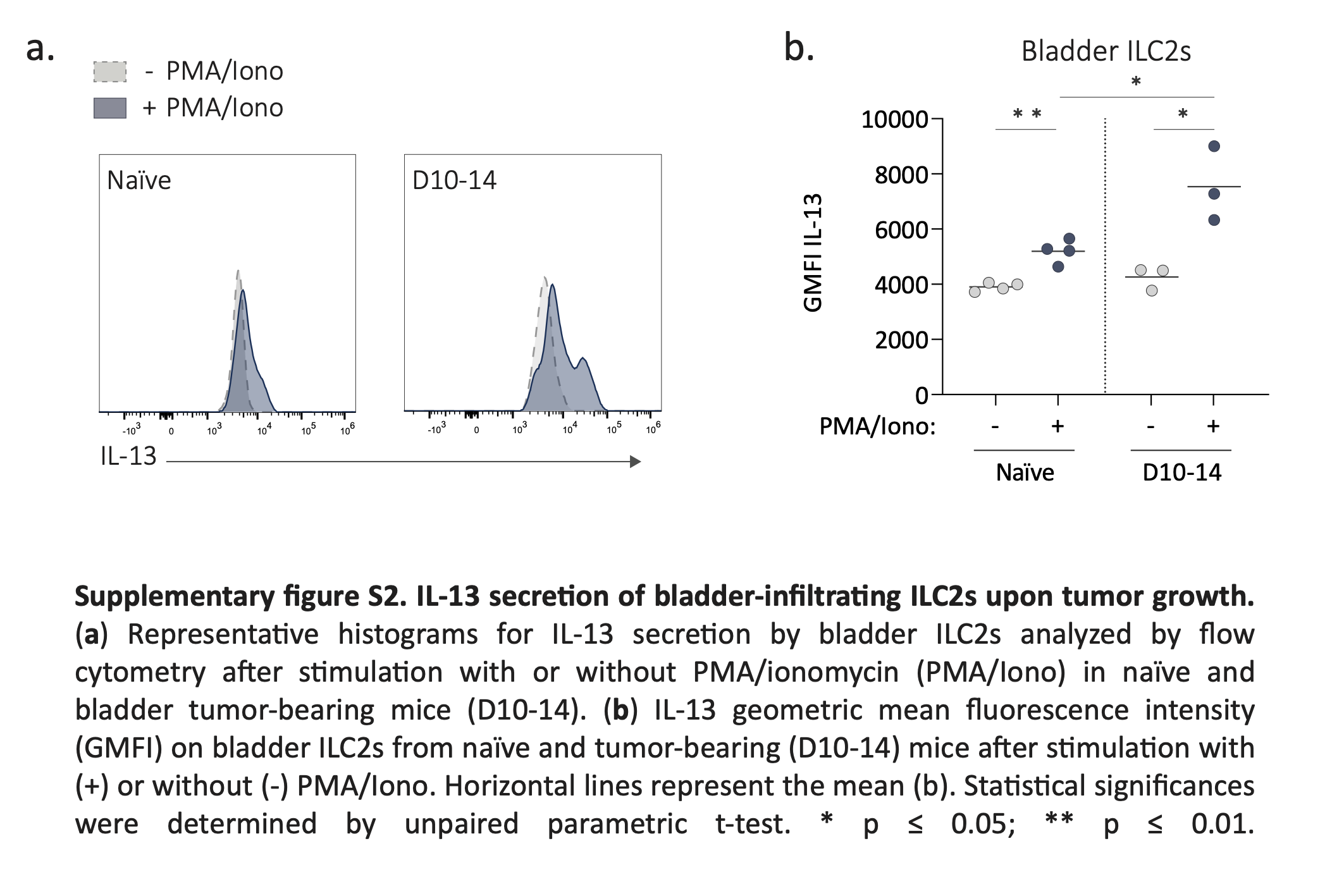

Supplement: Supplementary file 2 [file Image_2.tiff]

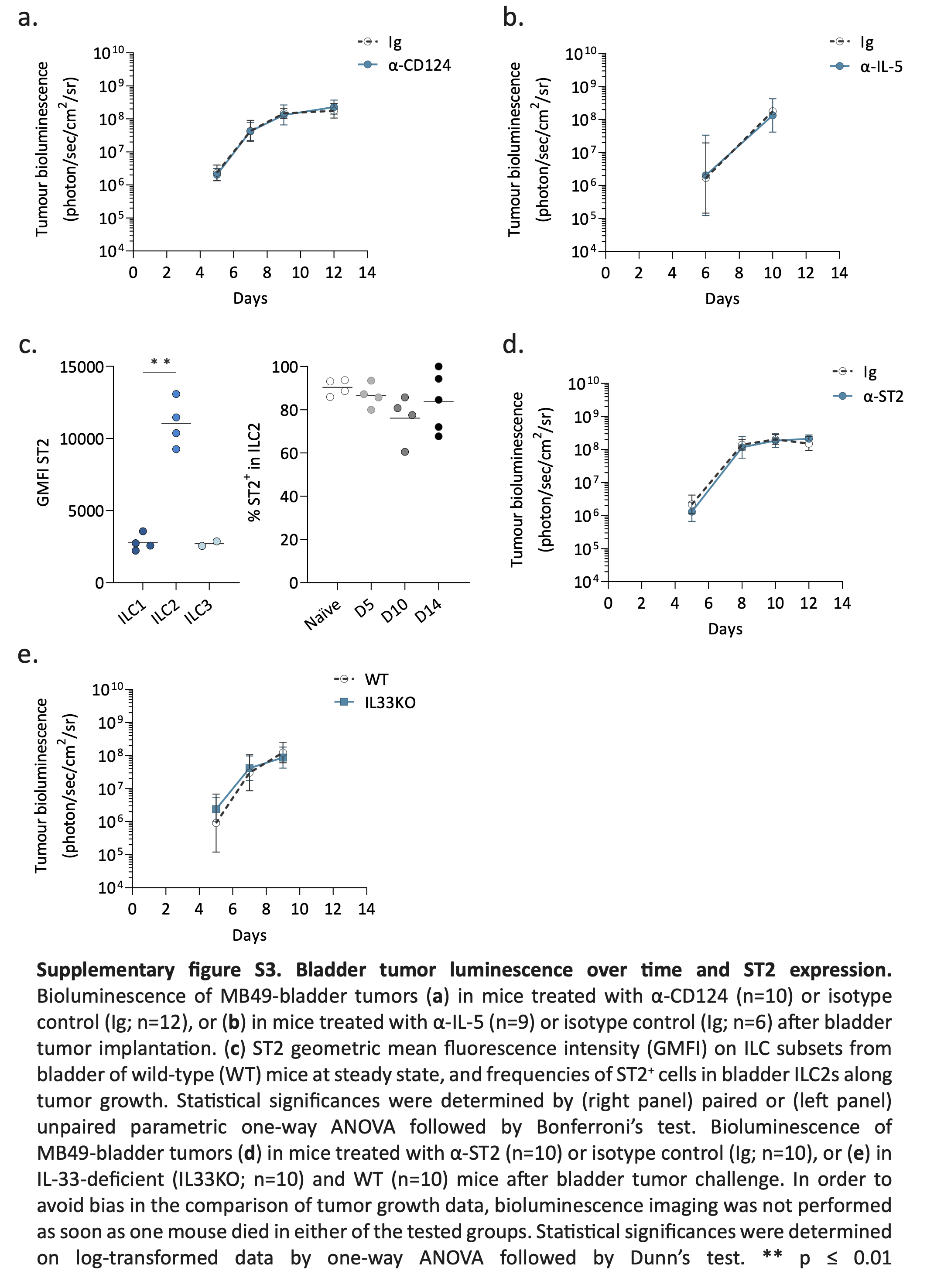

Supplement: Supplementary file 3 [file Image_3.tiff]

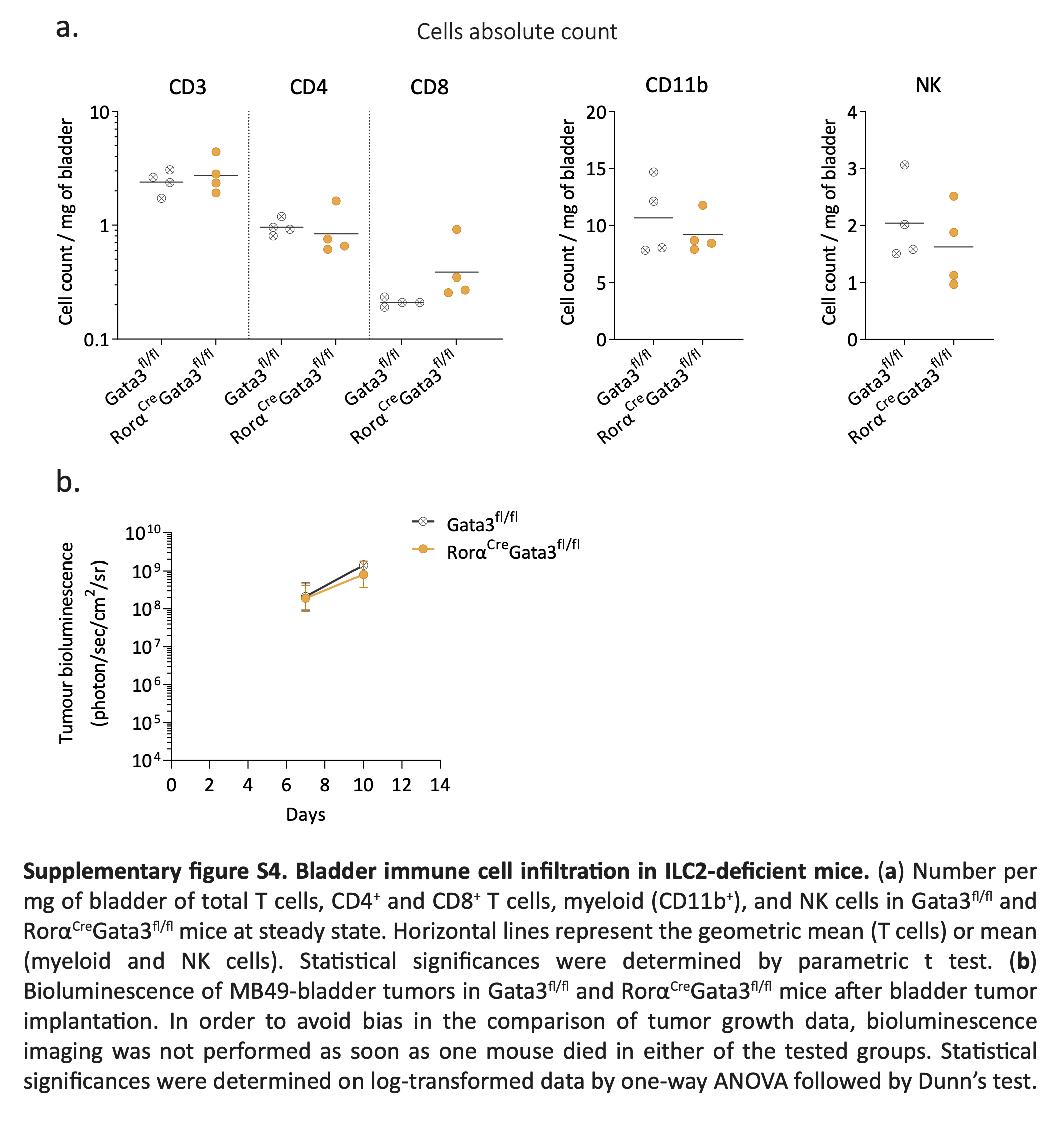

Supplement: Supplementary file 4 [file Image_4.tiff]
